# Renal hemosiderosis secondary to intravascular hemolysis after mitral valve repair: A case report

**DOI:** 10.1097/MD.0000000000018798

**Published:** 2020-01-17

**Authors:** In Hee Lee, Gun Woo Kang, Chang-Yeon Kim, Sun-Jae Lee, Min-Kyung Kim, Dong Jik Ahn

**Affiliations:** a Department of Internal Medicine; b Department of Pathology, Daegu Catholic University School of Medicine, Daegu; c Department of Pathology, Dongguk University College of Medicine, Gyeongju; d Department of Internal Medicine, Hansung Union Internal Medicine Clinic and Dialysis Center, Daegu, Republic of Korea.

**Keywords:** asymptomatic urinary abnormality, intravascular hemolysis, mitral valve repair, renal hemosiderosis

## Abstract

**Rationale:**

Renal hemosiderosis is a disease in which hemosiderin deposits in the renal cortex as a form of iron overload. However, cases of renal hemosiderosis due to intravascular hemolysis following mitral valve repair have been rarely reported.

**Patient concerns:**

We present the case of a 62-year-old woman who developed asymptomatic urinary abnormalities including microscopic hematuria and proteinuria due to renal hemosiderosis following a mitral valve repair surgery performed two years earlier.

**Diagnoses:**

A percutaneous renal biopsy showed no specific glomerular abnormality, tubular atrophy, or interstitial fibrosis but extensive deposition of hemosiderin in the proximal tubule. The patient was diagnosed with renal hemosiderosis and chronic intravascular hemolysis following mitral valve repair.

**Interventions:**

Our patient refused a mitral valve repeat surgery and hence was treated with oral iron preparations, *N-*acetylcysteine, and a β-receptor blocker.

**Outcomes:**

Moderate mitral regurgitation with the regurgitant blood striking against the annuloplasty ring was confirmed on follow-up echocardiography. After the 24-month follow-up period, hemolytic anemia persisted, but there was no significant decline of renal function.

**Lessons:**

For cases of chronic intravascular hemolysis accompanied with asymptomatic urinary abnormalities, a renal biopsy is required to exclude underlying kidney pathology and predict potential renal insufficiency.

## 1 Introduction

Renal hemosiderosis is a disease in which hemosiderin deposits in the renal cortex as a form of iron overload.^[1]^ This is often seen in various diseases with intravascular hemolysis such as paroxysmal nocturnal hemoglobinuria (PNH), mechanical hemolysis associated with severe valvular heart disease or cardiac valve surgery, and refractory anemia that needs frequent blood transfusion.^[2–4]^ In 1961, Sayed et al reported the onset of hemolysis in a patient with ostium primum atrial septal defect and an unrepaired mitral cleft.^[5]^ Since then, traumatic intravascular hemolysis has been considered one of the major complications of valvular heart disease or valve replacement surgery.^[6]^ However, cases of renal hemosiderosis due to intravascular hemolysis following mitral valve repair have been rarely reported.^[2,7]^ We report here, the case of a 62-year-old woman who developed renal hemosiderosis due to chronic intravascular hemolysis following a mitral valve repair surgery performed two years earlier for mitral regurgitation (MR) with atrial fibrillation.

## 2 Case report

**Figure 1:**
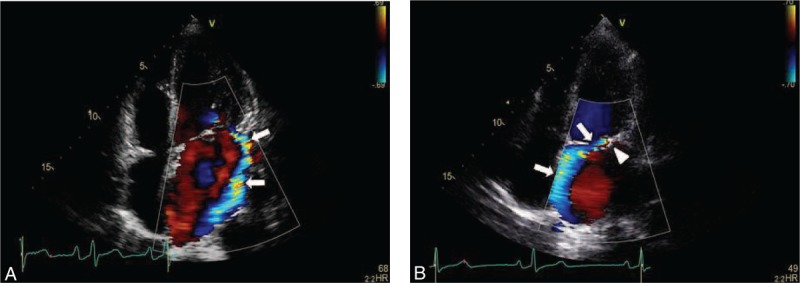
A: Color flow image of mitral regurgitant flow (arrows) before mitral valve repair. B: The reversed direction of mitral regurgitant flow (arrows) after surgery. The mechanism of hemolysis seems to be collision of the blood flow with the annuloplasty ring (arrowhead).

**Figure 2:**
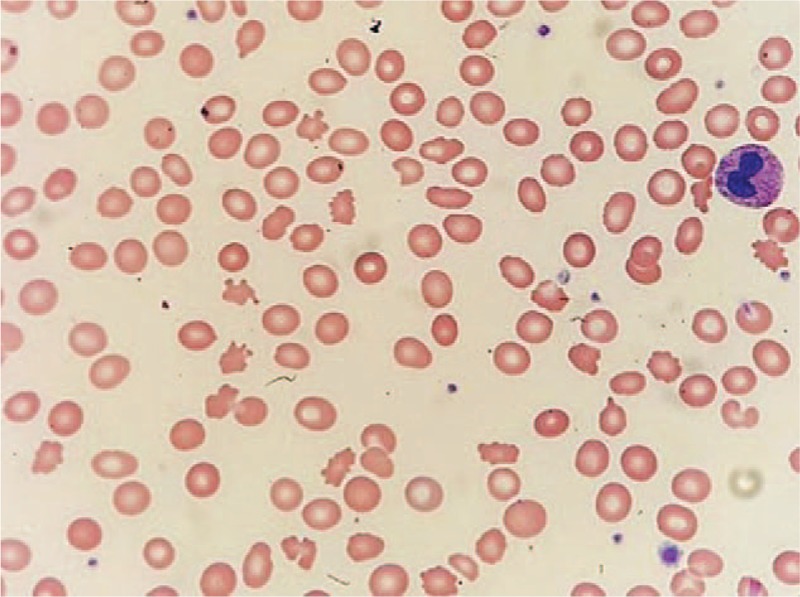
Peripheral blood smear showing fragmented red blood cells and schistocytes, suggestive of mechanical hemolysis.

**Figure 3:**
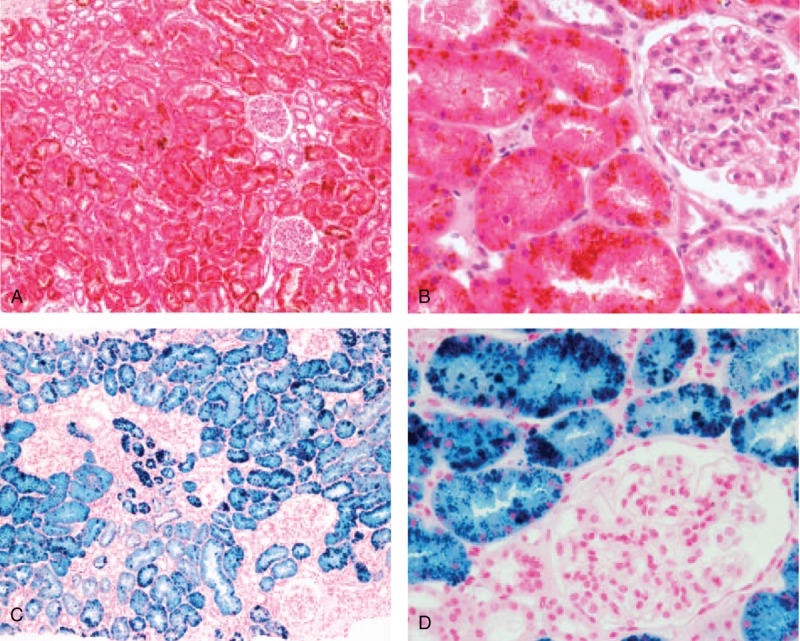
Light microscopy of renal biopsied specimen. (A, B) Hematoxylin and eosin (H & E) stains show extensive deposition of golden yellow to brown, coarsely granular pigments mostly within tubular epithelial cells. Glomerular architecture is normal. There is no evidence of acute tubular necrosis or tubulointerstitial nephritis (A: H & E stain, ×100), (B: H & E stain, ×400). (C-D) Prussian blue stains demonstrate widespread hemosiderin deposits of coarse blue granules in the tubules and the lumens (C: Prussian blue stain, ×100), (D: Prussian blue stain, ×400). H & E = Hematoxylin and eosin.

**Figure 4:**
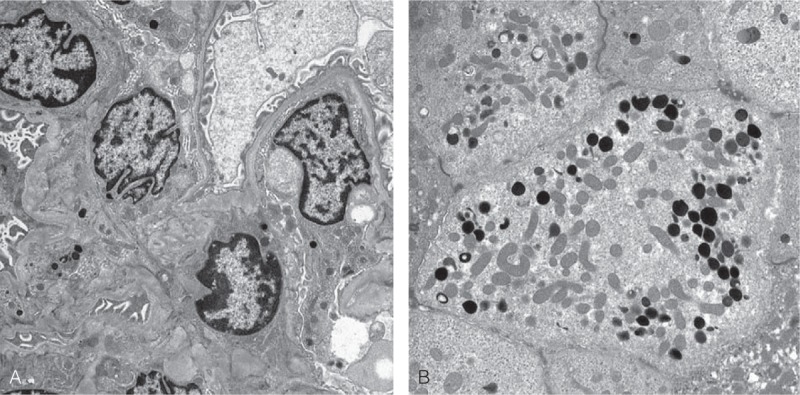
Electron microscopy of renal biopsied specimen. (A) Glomerular architecture is unremarkable, and there is no duplication of basement membrane, mesangial cell interposition, or electron-dense deposits in the mesangium and along the capillary walls (×7000). (B) Several lysosomes includes electron-dense particles within the cytoplasm of proximal tubular epithelial cells. The brush border and numerous mitochondria which are characteristic for the proximal tubular cell are also identified (×8000).

A 62-year-old female patient was referred to the nephrology unit for asymptomatic urinary abnormalities including microscopic hematuria and proteinuria, which had persisted for a year. The patient also complained of general weakness and mild dyspnea during exercise. Two years earlier, the patient had undergone mitral valve repair surgery for mitral regurgitation (MR) and paroxysmal atrial fibrillation with artificial chordae and an annuloplasty rings (Fig. 1A). During the current visit, the patient showed a blood pressure of 120/80 mmHg, pulse rate 65/min, respiration rate 22/min, and body temperature 36.5°C. Other than hyperthyroidism, she had no significant medical history such as diabetes mellitus, hypertension, or kidney disease; she also had no episodes of gross hematuria. Physical examination revealed conjunctival pallor but no evidence of jaundice in the sclerae. Moreover, there was no evidence of intra-abdominal organomegaly or edema of the lower limbs. Chest auscultation indicated regular heart sounds, with a holosystolic murmur (Grade IV/VI) in the apex region. A peripheral blood test at admission revealed the following: white blood cell (WBC) count, 4300/μL (neutrophils 59%); hemoglobin (Hb), 8.9 g/dL; and platelet count, 227,000/μL. Serum biochemical examination revealed the following: blood urea nitrogen (BUN), 24.1 mg/dL; creatinine (Cr), 0.8 mg/dL (estimated glomerular filtration rate, eGFR; 79 mL/min/1.73m^2^); aspartate aminotransferase, 55 IU/L; alanine aminotransferase, 16 IU/L; total protein, 6.8 g/dL; serum albumin, 4.3 g/dL; and C-reactive protein, 0.6 mg/L. Anemia-related hematological test results are described in Table 1. Urinalysis results were as follows: pH 5.5, occult blood 2+, and albumin 2+. Microscopic urinary sediment evaluation revealed 1–3 WBCs per high-power field (HPF) and 10–30 red blood cells (RBCs) per HPF (dysmorphic 80%). Twenty-four-hour urine examination revealed a urine protein level of 375 mg/day and a Cr clearance of 76.7 mL/min/1.73 m^2^. Serum immunoglobulin (Ig) levels, including IgG, IgA, and IgM, were normal, whereas the serum complement 3 (C3) level was reduced (67.2 mg/dL; normal range, 90–180 mg/dL); the levels of C4 and 50% hemolyzing dose of complement were normal. Moreover, serological test results for rheumatoid factor, viral markers (hepatitis B surface antigen, hepatitis C antibody, anti-human immunodeficiency virus antibody), lupus studies (antinuclear antibody, anti-double stranded DNA antibody), anti-neutrophil cytoplasmic antibody, and cryoglobulin were negative. On the chest radiograph, there were no abnormal findings, except for a slight cardiomegaly. The size and shape of both kidneys were normal on renal ultrasonography, whereas peripheral blood smear showed normocytic normochromic anemia with polychromasia, poikilocytosis, schistocytes, and spherocytes, suggesting hemolytic anemia (Fig. 2). PNH was excluded based on negative results of the flow cytometry tests for percentages of CD59+ cells and CD55+ cells among RBCs and granulocytes. Consequently, we suspected an asymptomatic urinary abnormality and performed a percutaneous renal biopsy on the third day after admission, to rule out glomerulopathy associated with a systemic disease. The renal biopsy showed 2 global glomerulosclerosis in all 23 glomeruli. The other glomeruli did not show significant pathologic abnormalities such as endocapillary proliferation, crescents or intravascular fibrin thrombi. However, interstitium showed extensive deposition of coarsely granular pigments mostly in tubular epithelial cells and within the tubular lumen on hematoxylin and eosin stains (Fig. 3A and B). Prussian blue iron stain demonstrated marked renal hemosiderosis with extensive iron depositions in the tubules (Fig. 3C and D). Immunofluorescence studies did not show any immune deposits in glomeruli. Electron microscopy showed unremarkable glomerular structure with no electron-dense deposits in the mesangium and along the capillary walls (Fig. 4A). However, irregularly shaped electron-dense particles were found within the lysosomes in tubules, particular in proximal tubular epithelial cells (Fig. 4B). Transthoracic echocardiography performed on the seventh day after admission showed moderate MR, which was due to tethering of the posterior leaflet. However, unlike in an MR due to typical posterior leaflet tethering, the direction of regurgitant blood flow was anterior, due to the blood striking the annuloplasty ring (Fig. 1B). On the basis of the laboratory and renal biopsy findings, the patient was diagnosed with chronic intravascular hemolysis and renal hemosiderosis following mitral valve repair. Subsequently, we recommended a mitral valve repeat surgery, but the patient refused surgery. We initiated a treatment regimen that included daily oral administration of ferrous sulfate 160 mg/day, *N-*acetylcysteine 200 mg/day, and bisoprolol 2.5 mg/day. Concentrated RBC transfusion was not performed. At 24 months after the kidney biopsy, renal function remained stable with biochemical test results showing BUN level of 19.4 mg/dL and Cr level of 0.9 mg/dL (eGFR; 76 mL/min/1.73m^2^); however, clinical signs of hemolytic anemia persisted (Table 1). Urinalysis results showed albumin 1+, RBC 5–10/HPF, and random urine protein-Cr ratio of 215 mg/g, which represented no significant differences compared to the results seen during the renal biopsy.

**Table 1:**
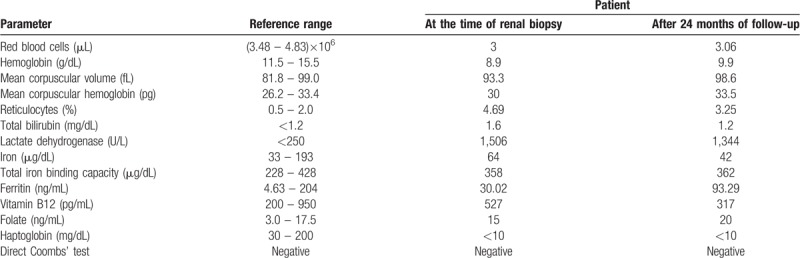
Patient's laboratory data of anemia studies.

## 3 Discussion

Mitral valve repair is the preferred treatment for severe MR, as it offers better results than valve replacement in terms of surgery-related mortality and survival rates as also superior preservation of the left ventricular function and fewer valve-related complications.^[8–10]^ However, recurrence of moderate or severe MR is seen after mitral valve repair, which can cause serious mechanical hemolysis. Hemolysis indicates a failure of the mitral valve repair procedure, and it is the second most common indication for repeat surgery, following suture dehiscence.^[9]^

When intravascular hemolysis occurs, free hemoglobin is released into the plasma and binds rapidly to haptoglobin. The hemoglobin-haptoglobin complex is degraded by the reticuloendothelial system. If hemolysis continues, the amount of free hemoglobin increases to saturate haptoglobin in the plasma, and subsequently, excess free hemoglobin in plasma is filtered through the glomeruli into the urinary space. After filtration, it is reabsorbed in the proximal tubule or detected in urine in the form of hemoglobinuria. Hemoglobin in the proximal tubule is degraded in the tubular epithelial cells, whereas free chelatable iron is stored in lysosomes in the form of hemosiderin.^[11]^ Chemically activated iron in hemosiderin can cause tubular injury through various mechanisms, such as free radical formation and lipid peroxidation, which may ultimately lead to tubular necrosis.^[2]^ Repeated or chronically persistent hemolysis can cause increased hemosiderin deposition, which can lead to irreversible structural changes such as tubular atrophy or interstitial fibrosis.^[12]^

So far, there have been two reported cases of intravenous hemolysis following mitral valve repair, with acute tubular injury and renal hemosiderosis detected on subsequent kidney biopsy.^[2,7]^ The patients in these cases presented with dark-colored urine and elevated serum Cr level at 3 and 7 months after mitral valve repair, respectively. Both patients showed normal glomeruli on renal biopsy, but tubular atrophy and interstitial fibrosis, along with extensive hemosiderin deposition in the tubules were noted. Both patients did not undergo repeat surgery and were administered with medications or multiple blood transfusions. During the follow-up period, their hemoglobin and serum Cr levels did not recover to the preoperative baseline levels. In contrast, the patient in our case was referred to the nephrology unit, two years after undergoing mitral valve repair, for persistent microscopic hematuria and non-nephrotic proteinuria. Renal biopsy showed no evidence of pathologic changes suggestive of acute tubular necrosis, tubular atrophy, or interstitial fibrosis (Fig. 3). Additionally, there was no significant decline in renal function during the subsequent 24-month follow-up period (Table 1). Therefore, we presume that damage to the renal tissue, especially the tubulointerstitial region, might be helpful in predicting future renal impairment in patients with renal hemosiderosis.

If a decline in hemoglobin or anemia persists after mitral valve repair, hematologic evaluation is necessary to detect intravascular hemolysis.^[13]^ A study of 32 patients with hemolysis after mitral valve repair, reported that hemolytic anemia appeared usually at an average of 3 months following surgery (range from 1 week to 4 years). Their mean hemoglobin level was 8.9 ± 1.3 g/dL at the time of diagnosis, with lactate dehydrogenase (LDH) level of 1,834 ± 1,505 U/L and a reticulocyte percentage of 4.3% ± 3.9%.^[14]^ During admission, our patient showed hemoglobin and LDH levels of 8.9 g/dL and 1,506 U/L, respectively, whereas her reticulocyte percentage was 4.69%, which are similar to the values in the other study mentioned above. These abnormal blood levels, with decreased plasma haptoglobin level and presence of schistocytes in the peripheral blood smear, satisfied all clinical diagnostic criteria for intravascular hemolysis suggested in a study by Skoularigis et al.^[15]^

Follow-up echocardiography after mitral valve repair can be very useful for assessing the degree of valvular abnormalities such as severity of MR and the mechanism for hemolysis. A study that performed echocardiography after mitral valve repair reported that all patients who showed clinically significant hemolysis had residual or recurrent MR. Of these, 77% showed moderate to severe regurgitation.^[14]^ Among the mechanisms for hemolysis after mitral valve repair, fragmentation (11/32, 34%) and rapid acceleration (10/32, 31%) were frequently found, while 13% (4/32) of the cases indicated collision jet that causes high shear stress, as in our patient.^[14]^ In our patient, moderate recurrent MR was observed, which involved posterior leaflet tethering. However, unlike most MR caused by posterior leaflet tethering, the regurgitation was directed anteriorly owing to the forceful abutment with the annuloplasty ring (Fig. 1B).

Once a patient is diagnosed with hemolytic anemia after mitral valve repair, optimal treatment is necessary, considering the clinical symptoms and laboratory and echocardiographic findings. If anemia is not severe, an adjuvant therapy using iron, folic acid, and vitamin B12 might help to relieve the symptoms.^[16]^ Antioxidants or medications that can improve RBC flexibility and reduce hydrodynamic shear forces can be used to treat hemolysis that occurs after cardiac valve surgery.^[2]^ In a previous case report, *N-*acetylcysteine was administered to patients with renal hemosiderosis accompanied by acute kidney injury, based on its pharmacological mechanism that reduces oxidative stress.^[17]^ In addition, other studies have reported improvement in hemolytic anemia by administration of pentoxifylline or β-adrenergic receptor blockers on a small number of patients.^[18–20]^ However, in patients who show persistent hemolysis and severe symptoms of anemia, requiring repeated RBC transfusions, or for those with exacerbation of residual mitral regurgitation despite medical treatments, immediate repair surgery is recommended.^[13]^ In our case, the patient refused surgery and consequently, we administered iron supplements, *N-*acetylcysteine, and low dose of β-receptor blocker. After 24 months of medical therapy, the clinical symptoms of the patient did not deteriorate, but laboratory findings suggesting chronic hemolysis persisted. Moreover, since it has been reported that the frequency of renal failure is higher in patients with renal hemosiderosis when the duration of hemolysis is longer or the degree of iron deposition is more compared to those with no deposition of iron,^[12]^ the renal function of our patient is being closely monitored and surgical revision for the valvular anomaly is still being considered.

In summary, we found evidence of chronic intravascular hemolysis and renal hemosiderosis in a 62-year-old female patient who had undergone mitral valve repair for MR and atrial fibrillation 2 years before presentation. Considering the findings in our case, it is important to suspect mechanical hemolysis caused by recurrent MR if severe anemia occurs and persists after mitral valve repair and to perform timely hematologic evaluation and follow-up echocardiography. Moreover, in cases of chronic intravascular hemolysis accompanied by asymptomatic urinary abnormalities, a renal biopsy is required to exclude underlying kidney pathology and predict potential renal insufficiency.

## Author contributions

**Conceptualization:** In Hee Lee.

**Data curation:** Gun Woo Kang, Chang-Yeon Kim.

**Formal analysis:** In Hee Lee, Sun-Jae Lee.

**Methodology:** In Hee Lee.

**Validation:** Min-Kyung Kim, Dong Jik Ahn.

**Writing – original draft:** In Hee Lee.

**Writing – review & editing:** In Hee Lee.

In Hee Lee orcid: 0000-0003-3562-7586.
